# Anticoagulant effects of edoxaban in cancer and noncancer patients with venous thromboembolism

**DOI:** 10.1186/s12959-025-00720-0

**Published:** 2025-04-16

**Authors:** Masashi Yoshida, Kentaro Ejiri, Naoaki Matsuo, Takanori Naito, Kazuhiro Kuroda, Koji Tokioka, Kunihiko Hatanaka, Ryohei Fujimoto, Hidenaru Yamaoka, Yutaka Kajikawa, Kazuki Suruga, Hiroki Sugiyama, Tsuyoshi Miyaji, Yoshimasa Morimoto, Nobuhiro Okamura, Toshihiro Sarashina, Satoshi Akagi, Toru Miyoshi, Kazufumi Nakamura, Hiroshi Ito, Shinsuke Yuasa

**Affiliations:** 1 Department of Cardiovascular Medicine, Okayama University Faculty of Medicine, Dentistry and Pharmaceutical Sciences, 2-5-1 Shikata-cho, Kita-ku, Okayama, Okayama 700- 8558 Japan; 2 Department of CKD and CVD, Okayama University Faculty of Medicine, Dentistry and Pharmaceutical Sciences, 2-5-1 Shikata-cho, Kita-ku, Okayama, Okayama 700-8558 Japan; 3 Department of General Internal Medicine 3, Kawasaki Medical School, 577 Matsushima, Kurashiki, Okayama 701-0192 Japan; 4 Department of Cardiovascular Medicine, Japanese Red Cross Okayama Hospital, 2-1-1 Aoe, Kita-ku, Okayama, Okayama 700-8607 Japan; 5 Department of Cardiovascular Medicine, Okayama City Hospital, 3-20-1 Kitanagaseomote-cho, Kita-ku, Okayama, Okayama 700-8557 Japan; 6 Department of Cardiovascular Medicine, Japanese Red Cross Society Himeji Hospital, 1-12-1 Shimotemo, Himeji, Hyougo 670-8540 Japan; 7 Department of Cardiovascular Medicine, Tsuyama Chuo Hospital, 1756 Kawasaki, Tsuyama, Okayama 708-0841 Japan; 8 Department of Cardiovascular Medicine, Okayama Rosai Hospital, 1-10-25 Chikkoumidorimachi, Nimani-ku, Okayama, Okayama 702-8055 Japan; 9 Department of Cardiovascular Medicine, NHO Fukuyama Medical Center, 4-14-17 Okinogami- cho, Fukuyama, Hiroshima 720-8520 Japan; 10 Department of Cardiovascular Medicine, Okayama Medical Center, 1711-1 Tamasu, Kita-ku, Okayama, Okayama 701-1192 Japan; 11 Department of Cardiovascular Medicine, Okayama Saiseikai General Hospital, 2-25 Kokutai- cho, Kita-ku, Okayama, Okayama 700-8511 Japan; 12 Hosogi Hospital, 37 Daizen-cho, Kochi, Kochi 780-8535 Japan; 13 Department of Cardiovascular Medicine, Fukuyama City Hospital, 5-23-1 Zaou-cho, Fukuyama, Hiroshima, 721-8511 Japan; 14 Okamura Isshindow Hospital, 2-1-7 Saidaijiminami, Higashi-ku, Okayama, Okayama 704-8117 Japan; 15 Kuroda Clinic, 2-8-35 Kanda-cho, Kita-ku, Okayama, Okayama 700-0935 Japan

**Keywords:** Factor Xa inhibitors, Anticoagulation effects, Cancer, Venous thromboembolism

## Abstract

**Background:**

Edoxaban, a direct oral anticoagulant (DOAC), is a first-line treatment for venous thromboembolism (VTE) and the suppression of VTE recurrence. In patients with cancer, however, recurrent VTE after DOAC treatment may be more common than in noncancer patients. To evaluate our hypothesis that the anticoagulation effect of edoxaban is lower in VTE patients with cancer than in noncancer patients.

**Methods:**

This study was a prospective, multicenter, observational study including patients treated with edoxaban for VTE in Japan. The primary outcome was the difference in the prothrombin time (PT), activated partial thromboplastin time (APTT), and D-dimer level at 5 h after initial edoxaban administration between the cancer and noncancer groups. An additional outcome was the longitudinal change in PT and APTT from 5 h to overnight after edoxaban administration. The incidence of adverse events was further investigated.

**Results:**

PT and APTT at 5 h after initial edoxaban administration were not significantly different between the cancer (*n* = 84) and noncancer groups (*n* = 138) (e.g., log-transformed APTT 3.55 vs. 3.55, *p* = 0.45). However, D-dimer in the cancer groups was significantly greater than that in the noncancer groups (log-transformed 1.83 vs. 1.79, *p* = 0.009). PT and APTT significantly decreased from 5 h to overnight after edoxaban, but a similar pattern was observed in each group. All adverse events after edoxaban administration were also similar between patients with cancer and noncancer.

**Conclusion:**

PT and APTT after edoxaban administration were similar between VTE patients with cancer and noncancer groups, suggesting that edoxaban has anticoagulation effects on cancer-associated VTE similar to those of noncancer patients.

**Trial registration:**

UMIN000041973; Registration Date: 2020.10.5.

## Introduction

Venous thromboembolism (VTE) is a preventable but potentially fatal disease. It is one of the main causes of death among hospitalized patients and the third most common cause of cardiovascular death [1]. VTE is common in cancer patients, with a 9-fold higher incidence rate than in noncancer patients [2]. Cancer patients with VTE have a poor prognosis; VTE is the second leading cause of death after cancer in cancer patients receiving outpatient chemotherapy [2–4]. To treat VTE and suppress VTE recurrence, anticoagulant therapies have been established [5–9]. Despite anticoagulant therapy, the VTE recurrence rate is high, and bleeding is a problem in cancer patients [10–12]. 

As anticoagulant therapy, vitamin K antagonists, heparins, and direct oral anticoagulants (DOACs) are available for VTE treatment. To treat VTE in cancer patients, the use of heparins, especially low-molecular-weight heparins (LMWH) and DOACs, is recommended [7–9]. DOACs are a common therapy in Japan because of the limited use of LMWH. The anticoagulant effects of DOACs are not monitored because of less individual variability in their pharmacological effects and short half-lives [13–15]. The anticoagulant effects of edoxaban, a DOAC, can be monitored by the prothrombin time (PT) and activated partial thromboplastin time (APTT) because the plasma concentration of edoxaban is correlated with the PT and APTT in healthy adults [16]. PT and APTT are prolonged up to 5 h after edoxaban administration [17]. 

The mechanisms of VTE in cancer patients include patient-related, cancer-related, and treatment-related factors. [18–20] Focusing on cancer-related factors, tissue factors, mucins, and other distinct cancer procoagulant factors that directly stimulate factor Xa cause a hypercoagulable state [21]. Hypercoagulability detected by a shorter APTT is associated with VTE [22]. APTT decreases after chemotherapy in breast cancer patients [23]. Therefore, we hypothesized that the anticoagulant effects of edoxaban are inhibited in cancer patients and measured the coagulable status by PT and APTT to assess its anticoagulant effects on VTE.

## Methods

### Study participants

The study of the anticoagulant effects of edoxaban in cancer patients and noncancer patients with venous thromboembolism (EVE study) is a prospective observational study conducted at 12 hospitals from July 2021–December 2022. This study was conducted in accordance with the principles of the Declaration of Helsinki and local regulations. The study protocol was reviewed and approved by the ethics committees of all the institutes. Data were collected via an electronic case report form (MID, Inc. Fukuoka, Japan). This study was registered at the UMIN Clinical Trials Registry (UMIN000041973).

Patients with newly diagnosed VTE receiving edoxaban for treatment were eligible for inclusion. VTE was diagnosed by imaging tests (lower-limb venous ultrasound and/or contrast-enhanced computed tomography). Patients were not eligible if they met any of the following exclusion criteria: were younger than 20 years old; had hemodynamical instability; were treated with thrombolytic therapy or thrombectomy; had advanced cancer and were expected to have a life expectancy of less than 3 months; were taking direct oral anticoagulants 48 h before initial edoxaban administration; had a continuous infusion of unfractionated heparin 3 h before initial edoxaban administration; had a subcutaneous infusion of enoxaparin 12 h before initial edoxaban administration; had a subcutaneous infusion of fondaparinux 24 h before initial edoxaban administration; had active bleeding or high risk of bleeding; had a risk of serious complications due to bleeding; had an uncontrolled blood pressure; had a coagulation disorder; had a cirrhosis; and were pregnant; had an acute bacterial endocarditis and creatinine clearance of < 15 mL/min. After determination of the treatment protocol, written informed consent was obtained before the initial blood test.

### Study protocol and study outcomes

**Fig. 1:**
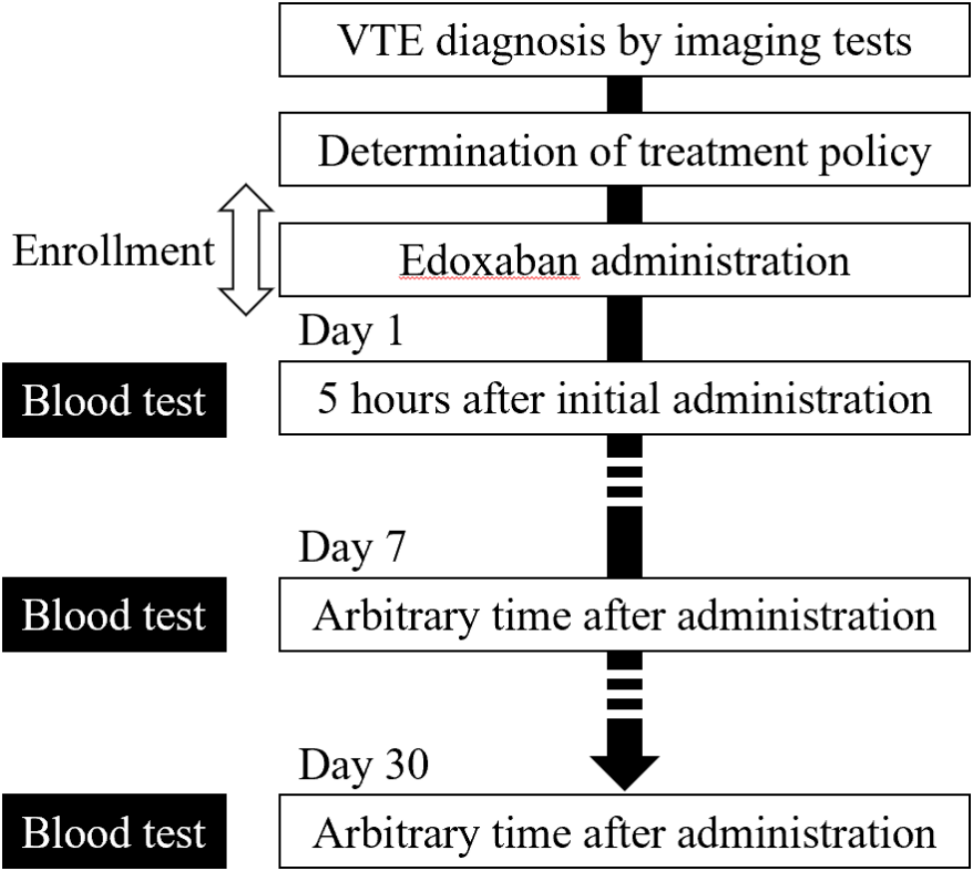
Overview of study protocol. Patients with newly diagnosed VTE with imaging tests receiving edoxaban were enrolled. Three blood tests were performed: 5 h after initial edoxaban administration and arbitrary time at approximately 7 and 30 days after administration. VTE, venous thromboembolism

The study design is shown in Fig. 1. After appropriate initial treatment in the acute phase, patients were orally administered edoxaban (30 or 60 mg) (Daiichi Sankyo Co., Ltd., Tokyo, Japan) tablets daily on the basis of their weight and renal function. The edoxaban dosage was reduced to 30 mg in patients who met any of the following criteria: body weight ≤ 60 kg, creatinine clearance ≤ 50 mL/min, or combined use with P-glycoprotein inhibitors. Blood samples were collected 3 times: 5 h after initial edoxaban administration and arbitrary time at approximately 7 and 30 days after initial edoxaban administration. The time of blood collection after the latest edoxaban administration was recorded by the attending physician. Blood samples were collected in tubes with 3.2% sodium citrate. To eliminate interinstitutional bias on PT, APTT, and D-dimer, blood plasma was frozen and measured at an independent central study laboratory (SRL, Inc., Tokyo, Japan). Blood tests other than coagulation tests (complete blood count and liver and kidney function tests) were performed at each institute. Patient information on demographics and medical history before edoxaban administration was obtained from all patients. Patient information on death, recurrent VTE, bleeding and discontinuation of edoxaban until 30 days after edoxaban administration was also obtained for all patients. The study participants were divided into two groups (the cancer and noncancer groups), and then, we compared the study outcomes between them.

The primary outcomes were PT, APTT, and D-dimer at 5 h after initial edoxaban administration in the cancer and noncancer groups. As a secondary outcome, to clarify the longitudinal change in coagulation after edoxaban administration, we assessed the differences in PT and APTT as coagulation parameters between 5 h and overnight after edoxaban administration in the cancer and noncancer groups. Additionally, we investigated all-cause death, recurrent VTE, bleeding, and the discontinuation of edoxaban as adverse events. Recurrent VTE was defined as symptomatic recurrence of deep vein thrombosis (DVT) or pulmonary embolism (PE), and asymptomatic DVT or PE were accidentally detected by imaging tests (computed tomography or echo) for diseases other than symptomatic DVT and PE. Bleeding was defined as major or clinically relevant nonmajor bleeding according to previous methods [24]. Major bleeding was defined if it was overt and was associated with a decrease in hemoglobin of 2 g per deciliter or more or if it required a transfusion of 2 or more units of blood, occurred at a critical site, or contributed to death. Clinically relevant nonmajor bleeding was defined as overt bleeding that did not meet the criteria for major bleeding but was associated with the need for medical intervention, contact with a physician, interruption of the study drug, or discomfort or impairment of activities of daily life. The occurrence of death was examined from the initiation of drug administration to 30 days after drug administration. Discontinuation of edoxaban was defined as the interruption of edoxaban for any reason.

### Covariates

Patient information related to VTE risk factors was collected for all patients [21, 25]. VTE risk factors include patient comorbidities, obesity (body mass index ≥ 25 kg/m^2^), underweight (body mass index < 18.5 kg/m^2^), immobilization longer than 4 days, infection, and surgery within 30 days. Patient comorbidities such as hypertension, dyslipidemia, diabetes, coronary artery disease, atrial fibrillation, cerebral infarction, and chronic obstructive pulmonary disease (COPD) were defined as a documented medical history for each disease. VTE risk factors related to cancer were diagnosed within 6 months, stage of cancer, presence of metastasis, and type of treatment (surgery, chemotherapy, and radiation). The cancer patients were those who were diagnosed within 6 months or who were receiving cancer treatment. Candidate biomarkers for VTE risk are the leukocyte count, hemoglobin level, platelet count, creatinine clearance, and C-reactive protein (CRP) level.

### Statistical analysis

Analysis was performed with the exclusion of patients who did not meet the inclusion criteria, withdrew consent, and whose data were collected incompletely.

We compared patient characteristics between the cancer and noncancer groups. Continuous variables are presented as medians (interquartile ranges: IQRs), and categorical variables are summarized as counts and proportions (%).

For primary outcomes, PT, APTT, and D-dimer were log-transformed for reliable analyses, and then, we ran analysis of covariance (ANCOVA) to estimate differences (i.e., β coefficients) and 95% confidence intervals (CIs) between the cancer and noncancer groups. For potential confounders, we adjusted for age, male sex, hypertension, dyslipidemia, diabetes, obesity, history of surgery within 30 days, infection, immobilization longer than 4 days, leukocyte count (log-transformed), hemoglobin, platelet, creatinine clearance, CRP (log-transformed), history of cancer surgery, chemotherapy, radiation therapy, history of coronary artery disease, atrial fibrillation, stroke, and COPD. The same analytic approach applied in subgroups stratified by the dose of edoxaban (30 mg vs. 60 mg) and the interaction between edoxaban dose and outcomes was tested via the likelihood ratio test.

To assess the longitudinal changes in coagulation parameters after edoxaban administration, we modeled a mixed-effect linear regression model (random intercept model) adjusted for the same confounders above with a compound symmetry correlation matrix taking into account the within-patient correlation of each measure. The changes (i.e., β coefficient [slope]) and 95% CIs in PT and APTT from 5 h to overnight after edoxaban administration were separately assessed in each group (cancer and noncancer). We further evaluated whether cancer modified the pattern of change in coagulation parameters via a likelihood ratio test for models with or without an interaction term.

Additionally, we investigated the incidence of adverse events after edoxaban administration, including all-cause death, the incidence of recurrent VTE, bleeding, and the discontinuation of edoxaban. *P* < 0.05 was considered to indicate statistical significance. All *P* values are 2-sided. All analyses were performed via JMP ver. 11.0 (SAS Institute, Cary, NC, USA) and R version 4.2.3 (The R Foundation for Statistical Computing, Vienna, Austria).

## Results

### Study flow and baseline characteristics

**Fig. 2:**
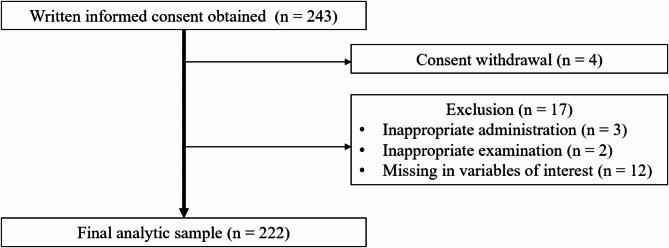
A flow diagram of the study. A total of 222 participants were analyzed out of 243 participants

Two hundred forty-three patients were enrolled for study screening (Fig. 2). Four patients who withdrew from the study were excluded. Five patients who did not meet the inclusion criteria and/or met the exclusion criteria were excluded. Twelve patients were excluded because of lack of data, and a total of 222 patients were included in the present study.

The median age was 75 years (IQR 67–82) (Table 1). In the overall population, ninety-three patients (42%) were male, 82 (37%) received edoxaban 60 mg, and 84 (38%) had cancer. Sixty-four patients were obese (29%), 91 underwent surgery within 30 days, 61 were immobile longer than 4 days (27%), and 27 had current infections (12%). The median leukocyte count, hemoglobin, platelet count, creatinine clearance, and CRP were 6.6 (IQR 5.0–8.8) ×10^3^/mL, 11.4 (10.0–13.0) g/dL, 242 (185–314) ×10^3^/mL, 64 (47–83) mL/min, and 1.3 (0.3–4.7) mg/dL, respectively. Overall, a similar pattern was observed for most variables between the cancer and noncancer groups. In the cancer group, male patients were more common than in the noncancer group (59% vs. 31%). Similarly, the other etiological risk factors for VTE other than cancer (e.g., obesity, surgery within 30 days, immobilization longer than 4 days, and infection) were more frequently observed in the noncancer group than in the cancer group. In terms of laboratory findings, the CRP level in the cancer group was greater than that in the noncancer group. In the cancer group, 53 (63%) and 38 (45%) patients were diagnosed with cancer within 6 months and with metastatic cancer, respectively. Among them, for cancer treatment, cancer surgery was the most common (63%), followed by chemotherapy (44%) and radiation (8%).

**Table 1:** Patient characteristics in cancer patients and noncancer patients

		Overall	Cancer	Noncancer
		(*n* = 222)	(*n* = 84)	(*n* = 138)
Age, years		75 (67–82)	75 (66–81)	75 (67–84)
Male (%)		93 (42)	50 (59)	43 (31)
Edoxaban 60 mg (%)		82 (37)	33 (39)	49 (36)
Symptom				
	Syncope (%)	5 (2)	2 (2)	3 (2)
	Dyspnea (%)	39 (18)	14 (17)	25 (18)
	Limb swelling (%)	40 (18)	11 (13)	29 (21)
Cardiovascular disease risk				
	Hypertension (%)	121 (55)	45 (54)	74 (53)
	Dyslipidemia (%)	66 (30)	27 (32)	39 (28)
	Diabetes (%)	42 (19)	15 (18)	27 (20)
	Coronary artery disease (%)	7 (3)	4 (5)	3 (2)
	Atrial fibrillation (%)	1 (1)	0 (0)	1 (1)
	Cerebral infarction (%)	7 (3)	4 (5)	3 (2)
	Chronic obstructive lung disease (%)	9 (4)	5 (6)	4 (3)
VTE risk				
	Obesity (%)	64 (29)	18 (21)	46 (33)
	Underweight (%)	26 (12)	10 (12)	16 (12)
	Surgery within 30 days (%)	91 (41)	31 (37)	60 (44)
	Immobilization longer than 4 days (%)	61 (27)	17 (20)	44 (32)
	Infection (%)	27 (12)	9 (11)	18 (13)
Laboratory findings				
	Leukocyte count, ×10^3^/mL	6.6 (5.0–8.8)	7.0 (5.0–10.6)	6.5 (5.1–8.1)
	Hemoglobin, g/dL	11.4 (10.0–13.0)	11.0 (8.8–12.6)	11.7 (10.1–13.3)
	Platelet count, ×10^3^/mL	242 (185–314)	235 (175–310)	242 (193–319)
	Aspartate Aminotransferase, U/L	23.0 (18.0–32.0)	24 (17.3–33.8)	22 (18.0-31.3)
	Alanine Aminotransferase, U/L	19.0 (13.0–29.0)	19 (12.3–30.1)	18 (13.0–28.0)
	Creatinine clearance, mL/min	64 (47–83)	64 (52–81)	63 (46–85)
	C-reactive protein, mg/dL	1.3 (0.3–4.7)	1.7 (0.5–6.9)	1.1 (0.2–3.6)
Cancer		84 (38)	84 (100)	NA
	Diagnosed within 6 months (%)		53 (63)	
	Stage of cancer (I/II/III/IV)		17/11/18/38	
	Metastasis (%)		38 (45)	
	Surgery (%)		53 (63)	
	Chemotherapy (%)		37 (44)	
	Radiation (%)		7 (8)	

Data are presented as medians (interquartile ranges: IQRs) or counts and proportions (%)

NA, Not applicable; VTE, venous thromboembolism

### Coagulation markers and cancer

**Fig. 3:**
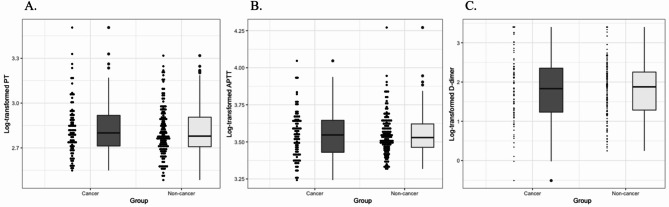
Comparison of coagulation parameters at 5 h after edoxaban treatment between cancer and noncancer groups. Each panel shows coagulation parameters at 5 h after edoxaban administration (**A**, PT; **B**, APTT; and **C**, **D**-dimer). The dots indicate the coagulation parameters of each individual. Box plots indicate the median and interquartile range of coagulation parameters between the cancer (dark gray) and noncancer (light gray) groups. Statistical testing was performed according to analysis of covariance adjusted for age, male sex, hypertension, dyslipidemia, diabetes, obesity, history of surgery within 30 days, infection, immobilization longer than 4 days, leukocyte count (log-transformed), hemoglobin, platelet, creatinine clearance, CRP (log-transformed), history of cancer surgery, chemotherapy, radiation therapy, history of coronary artery disease, atrial fibrillation, stroke, and COPD. PT, prothrombin time; APTT, activated partial thromboplastin time; CRP, C-reactive protein; and COPD, chronic obstructive pulmonary disease

The primary results of the present study are shown in Table 2; Fig. 3. Five hours after initial edoxaban administration, the PT in the cancer group was greater than that in the noncancer group, but it did not reach statistical significance (log-transformed PT 2.83 vs. 2.81; Δ0.071, 95% CI [− 0.005, 0.148]; *p* = 0.07). Similarly, the cancer group was not significantly associated with APTT compared with the noncancer group. For D-dimer, however, the cancer group showed a significant association (log-transformed D-dimer 1.83 vs. 1.79; Δ0.456, 95% CI [0.119, 0.792]; *p* = 0.009).

**Table 2:** Comparison of coagulation parameters at 5 h after Edoxaban administration between cancer and noncancer groups

Edoxaban 30 mg and 60 mg (*n* = 222)		Cancer (*n* = 84)	Noncancer (*n* = 138)	Difference (95% CI)	*p* value
PT	Median (IQR), second	16.5 (15–18.5)	16.1 (15–18.3)		
	Mean (SD) (Log-transformed)	2.83 (0.18)	2.81 (0.17)	0.071 (− 0.005, 0.148)	0.07
APTT	Median (IQR), second	34.7 (30.1–38.3)	34.1 (31.9–37.4)		
	Mean (SD) (Log-transformed)	3.55 (0.16)	3.55 (0.14)	0.025 (− 0.039, 0.091)	0.45
D-dimer	Median (IQR), µg/mL	6.2 (3.4–10.7)	6.5 (3.6–9.5)		
	Mean (SD) (Log-transformed)	1.83 (0.87)	1.79 (0.7)	0.456 (0.119, 0.792)	0.009

Differences (i.e., β coefficients) and 95% CIs were estimated via analysis of covariance

Models adjusted for age, male sex, hypertension, dyslipidemia, diabetes, obesity, history of surgery within 30 days, infection, immobilization longer than 4 days, leukocyte count (log-transformed), hemoglobin, platelet, creatinine clearance, CRP (log-transformed), history of cancer surgery, chemotherapy, radiation therapy, history of coronary artery disease, atrial fibrillation, stroke, and COPD

The interaction effects between edoxaban dose (30 mg or 60 mg) and patient outcome were tested via the likelihood ratio test

PT, prothrombin time; APTT, activated partial thromboplastin time; CRP, C-reactive protein; COPD, chronic obstructive pulmonary disease

After stratification by edoxaban dose (30 mg vs. 60 mg), compared with the overall population, similar results were observed in patients treated with edoxaban 30 mg. However, PT in the cancer group was significantly greater than that in the noncancer group (log-transformed PT 2.79 vs. 2.75; Δ0.135, 95% CI [0.056, 0.214]; *p* = 0.001, Table 3). In patients treated with 60 mg, on the other hand, cancer showed an inverse association with PT and D-dimer, and the associations between cancer and each coagulation marker were not statistically significant. A significant interaction of cancer (vs. noncancer) with PT and D-dimer was detected (p-for-interaction, *p* = 0.009 and 0.002, respectively).

**Table 3:** Coagulation parameters at 5 h after Edoxaban administration stratified by the dose of Edoxaban

Edoxaban 30 mg (*n* = 140)		Cancer (*n* = 51)	Noncancer (*n* = 89)	Difference (95% CI)	*p* value	*p* for interaction
PT	Median (IQR), second	16.2 (14.7–17.5)	15.7 (14.5–17.1)			
	Mean (SD) (Log-transformed)	2.79 (0.16)	2.75 (0.13)	0.135 (0.056, 0.214)	0.001	0.009
APTT	Median (IQR), second	32.9 (30.1–36.3)	32.8 (30.5–35)			
	Mean (SD) (Log-transformed)	3.51 (0.16)	3.51 (0.14)	0.062 (− 0.025, 0.149)	0.17	0.43
D-dimer	Median (IQR), µg/mL	7.27 (3.6–11.3)	5.76 (3.26–9.08)			
	Mean (SD) (Log-transformed)	1.88 (0.86)	1.71 (0.74)	0.884 (0.451, 1.317)	< 0.001	0.002
Edoxaban 60 mg (*n* = 82)		Cancer (*n* = 33)	Noncancer (*n* = 49)			
PT	Median (IQR), second	17.5 (16–19.1)	18.1 (16.1–21.1)			
	Mean (SD) (Log-transformed)	2.89 (0.2)	2.92 (0.18)	−0.015 (− 0.149, 0.119)	0.83	
APTT	Median (IQR), second	37.4 (34.2–39.4)	36.4 (34.3–39.9)			
	Mean (SD) (Log-transformed)	3.6 (0.14)	3.62 (0.13)	0.053 (− 0.018, 0.029)	0.17	
D-dimer	Median (IQR), µg/mL	5.33 (3.41–9.49)	6.98 (4.75–10.8)			
	Mean (SD) (Log-transformed)	1.76 (0.89)	1.94 (0.6)	−0.036 (− 0.578, 0.506)	0.9	

Differences (i.e., β coefficients) and 95% CIs were estimated via analysis of covariance

Models adjusted for age, male sex, hypertension, dyslipidemia, diabetes, obesity, history of surgery within 30 days, infection, immobilization longer than 4 days, leukocyte count (log-transformed), hemoglobin, platelet, creatinine clearance, CRP (log-transformed), history of cancer surgery, chemotherapy, radiation therapy, history of coronary artery disease, atrial fibrillation, stroke, and COPD

The interaction effects between edoxaban dose (30 mg or 60 mg) and patient outcome were tested via the likelihood ratio test

PT, prothrombin time; APTT, activated partial thromboplastin time; CRP, C-reactive protein; COPD, chronic obstructive pulmonary disease

### Longitudinal changes in coagulation markers

**Fig. 4:**
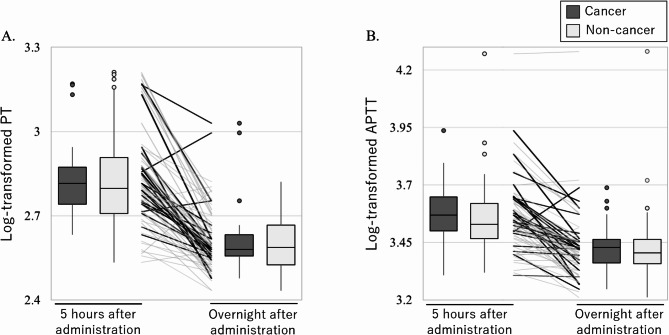
Longitudinal changes in PT and APTT between the cancer and noncancer groups. Each panel shows coagulation parameters at 5 h and overnight after edoxaban administration (**A**; PT and **B**; APTT). The line graph indicates longitudinal changes in coagulation parameters in each individual. Box plots indicate the median and interquartile range of coagulation parameters between the cancer (dark gray) and noncancer (light gray) groups

A total of 24 patients with cancer and 51 patients without cancer were included in the longitudinal analyses (Table 4; Fig. 4). PT and APTT significantly decreased from 5 h to overnight after edoxaban administration in both the cancer and noncancer groups (e.g., APTT, 3.58 vs. 3.43; β coefficient, − 0.151; 95% CI, [− 0.207, − 0.095]; *p* < 0.001; Table 4). With respect to the longitudinal changes in PT and APTT, a similar pattern was observed between the cancer and noncancer groups (no significant interaction).

**Table 4:** Longitudinal changes in PT and APTT between the cancer and noncancer groups

Coagulation markers		Cancer (*n* = 24)			Noncancer (*n* = 51)			
		Measurement timing			Measurement timing			
		Five hours later	Overnight	β coefficient (95% CI)	Five hours later	Overnight	β coefficient (95% CI)	*p* value for interaction
PT	Median (IQR), second	16.7 (15.6–17.6)	13.2 (12.9–13.8)		16.4 (15–18.2)	13.3 (12.5–14.3)		
	Mean (SD) (Log-transformed)	2.84 (0.14)	2.63 (0.14)	−0.214 (− 0.274, − 0.153)	2.82 (0.17)	2.6 (0.1)	−0.216 (− 0.258, − 0.175)	0.95
APTT	Median (IQR), second	35.5 (33.2–38.3)	30.8 (29.1–31.8)		34.1 (32–36.8)	30.1 (28.7–31.8)		
	Mean (SD) (Log-transformed)	3.58 (0.14)	3.43 (0.11)	−0.151 (− 0.207, − 0.095)	3.55 (0.16)	3.43 (0.16)	−0.125 (− 0.152, − 0.098)	0.36

The β coefficient and 95% CI were estimated via mixed effect linear regression models

The models were adjusted for age; male sex; hypertension; dyslipidemia; diabetes; obesity; a history of surgery within 30 days; infection; immobilization for more than 4 days; leukocyte count (log-transformed); hemoglobin; platelet count; creatinine clearance; CRP level (log-transformed); history of cancer surgery; chemotherapy; radiation therapy; and history of coronary artery disease, atrial fibrillation, stroke, and COPD

The interaction between cancer (vs. noncancer) and longitudinal changes (i.e., slope) was tested via the likelihood ratio test

PT, prothrombin time; APTT, activated partial thromboplastin time

### Adverse events in cancer and noncancer patients

Adverse events, including all-cause death, recurrent VTE, bleeding and the discontinuation of edoxaban, are shown in Table 5. All-cause death occurred in 8 patients (4%) due to progression of cancer or pneumonia, and VTE recurrence occurred in 4 (2%) overall patients. Major bleeding and clinically relevant nonmajor bleeding occurred in 5 patients (2%) and 6 (3%) overall. Discontinuation of edoxaban occurred in 20 patients because of VTE recurrence or bleeding. The proportion of each adverse event was similar between the cancer and noncancer groups.

**Table 5:** Adverse events in cancer and noncancer patients

		Overall	Cancer	Noncancer
		(*n* = 222)	(*n* = 84)	(*n* = 138)
Death		8 (4)	4 (5)	4 (2)
	VTE	0 (0)	0 (0)	0 (0)
	Cancer progression	4 (2)	4 (5)	NA
	Pneumonia	2 (1)	0 (0)	2 (1)
	Interstitial pneumonia	2 (1)	0 (0)	2 (1)
VTE recurrence		4 (2)	2 (2)	2 (1)
	PE	1 (1)	0 (0)	1 (1)
	DVT	3 (1)	2 (2)	1 (1)
Major bleeding		5 (2)	2 (2)	3 (2)
	Intracranial hemorrhage	1 (1)	0 (0)	1 (1)
	Muscular hemorrhage	1 (1)	0 (0)	1 (1)
	Retroperitoneal hemorrhage	1 (1)	1 (1)	0 (0)
	Gastrointestinal bleeding	2 (1)	1 (1)	1 (1)
Clinically relevant nonmajor bleeding		6 (3)	2 (2)	4 (3)
Discontinuation of edoxaban		20 (9)	10 (12)	10 (7)

The data are presented as counts and proportions (%)

NA, not applicable; VTE, venous thromboembolism; PE, pulmonary embolism; DVT, deep vein thrombosis

## Discussion

In a multicenter, prospective, observational study including cancer or noncancer VTE patients (40% were male) with initial edoxaban administration (40% were prescribed 30 mg), the PT and APTT at 5 h in the cancer group were greater than those in the noncancer group but did not reach statistical significance. Compared with the noncancer group, the cancer group had significantly higher D-dimer levels. A similar pattern was observed in patients treated with edoxaban 30 mg but not in those treated with edoxaban 60 mg, and the dose of edoxaban significantly affected the association. In both the cancer and noncancer groups, however, PT and APTT significantly decreased from 5 h to overnight after edoxaban administration. Additionally, the proportion of adverse events after edoxaban administration was similar between the cancer and noncancer groups.

In contrast to our hypothesis, the results of the present study suggest that the anticoagulant effects of edoxaban could be similar or even slightly stronger in cancer patients than in noncancer patients. A significant interaction effect according to the dose of edoxaban was detected in the association of cancer with coagulation parameters, although the mechanism is not clear. However, these results suggest that even low-dose edoxaban has similar effects on cancer and noncancer groups.

In this study, the dose reduction of edoxaban was based on appropriate use. Since cancer patients could suffer from weight loss and impaired renal function due to cancer progression, they probably meet the criteria of reducing dose of edoxaban than non-cancer patients. In this cohort, however, patients with a life expectancy of less than three months were excluded and there was no difference in the proportion of underweight between the cancer and noncancer groups. Renal function, as measured by creatinine clearance, also did not differ between the two groups. Therefore, we think that the dose of edoxaban did not affect the results of this study.

In previous studies, the anticoagulant effects of edoxaban were assessed on the basis of the plasma concentration of edoxaban approximately 2 h after administration, which was measured via liquid chromatography‒tandem mass spectrometry in healthy volunteers or cancer patients receiving particular drugs to determine its safety and drug‒drug interactions [12, 26, 27]. These reports revealed that prolonged PT and APTT were associated with edoxaban concentration. Compared with previous reports, the present study has several unique aspects. First, since the present study was a prospective, observational, clinical study, coagulation parameters were measured after edoxaban administration as a treatment in clinical practice rather than in experimental settings. To avoid intersample errors, since the anticoagulant effects of edoxaban last for at least 5 h [14, 17], we measured coagulation parameters 5 h after administration rather than 2 h after administration. Therefore, the results of the present study can be interpreted more pragmatically than those of previous reports.

Interestingly, a greater D-dimer level 5 h after edoxaban 30 mg administration was observed in the cancer group than in the noncancer group. In the noncancer group, however, other conventional risk factors for VTE (e.g., obesity, surgery within 30 days, immobilization longer than 4 days, and infection) were more frequently observed. Previous studies have demonstrated that cancer causes hypercoagulation through different mechanisms from conventional risk factors (e.g., specific cytokines) [18]. The findings of the present study suggest that hypercoagulation may remain even after edoxaban 30 mg administration in cancer patients. In contrast, there was no difference in D-dimer levels 5 h after edoxaban 60 mg administration between the cancer and noncancer group. A higher PT value with edoxaban 60 mg compared to 30 mg, indicating a stronger anticoagulant effect of edoxaban, may have counteracted the hypercoagulation caused by cancer, resulting in similar D-dimer levels between the cancer and noncancer groups. Although the incidence of recurrent VTE was similar between the cancer and noncancer groups, the results should be interpreted as exploratory because of the limited sample size.

One retrospective study among clinical trials of edoxaban for the treatment of VTE revealed that the efficacy and safety of edoxaban for the treatment of cancer-associated VTE are comparable to those of noncancer-associated VTE [28]. The findings of the present study are in line with previous findings. However, a few studies reported that DOAC treatments for patients with cancer tend to be associated with major bleeding compared with conventional LMWH treatment [21]. In particular, the presence of gastrointestinal cancer or the use of combination chemotherapy may be related to a high risk of bleeding after anticoagulation [26]; thus, clinicians should carefully monitor such patients, and further investigation is warranted.

The present study has several limitations. First, the present study was a multicenter, prospective, observational study with a small sample size. The number of study participants was limited; thus, low statistical power might have contributed to the results of this study. In particular, compared to the previous studies [24, 30], the lower event rate in the adverse outcome was observed in this study. Therefore, the comparison of the adverse event between cancer and non-cancer patients was not appropriate in this study. The findings of this study, therefore, should be interpreted as exploratory in nature. Second, because the present study included only Japanese individuals, the generalizability of the findings of the study to racial groups other than Asians should be limited. A subgroup analysis of the Hokusai VTE trial showed that East Asian patients had a similar VTE recurrence rate but a higher tendency for clinically relevant bleeding compared to non-East Asian patients; however, the safety and efficacy of edoxaban in VTE was consistent between East Asian and non-East Asian [31]. On the other hand, in the real-world setting, the difference in genetic, socio-economic status, and healthcare system among racial and ethnic diversity may affect the administration and effectiveness of anticoagulant therapy for VTE. Third, baseline PT and APTT (i.e., before edoxaban administration) were not available for analysis because of the study protocol. The study aims, however, was not to assess changes in coagulation parameters before and after the administration of edoxaban. Fourth, the PT and APTT at 5 h after edoxaban administration may not simply reflect the anticoagulation effect of edoxaban. Because the half-life of edoxaban is approximately 8–10 h [29], PT and APTT overnight after administration were measured as close to their trough concentrations. We further compared the effects of edoxaban in cancer patients and noncancer patients in terms of PT and APTT from 5 h to overnight after edoxaban administration. Finally, selection bias and residual confounding bias were inevitable in this type of observational study. To overcome these potential limitations, further multicenter prospective studies with larger sample sizes are warranted.

## Conclusions

In a multicenter, prospective, observational study including VTE patients, PT and APTT after initial edoxaban administration were not significantly different between the cancer and noncancer groups. Compared with noncancer patients, however, D-dimer levels after edoxaban were greater in the cancer group. The findings of this study suggest that edoxaban has a similar anticoagulation effect in both the cancer and noncancer groups, but hypercoagulation by cancer may persist even after edoxaban administration.

## Data Availability

No datasets were generated or analysed during the current study.

## Abbreviations

DOAC: Direct oral anticoagulant
VTE: Venous thromboembolism
PT: Prothrombin time
APTT: Activated partial thromboplastin time
LMWH: Low-molecular-weight heparin
COPD: Chronic obstructive pulmonary disease
CRP: C-reactive protein
